# Posicionamento do Departamento de Ergometria, Exercício, Cardiologia Nuclear e Reabilitação Cardiovascular (DERC/SBC) sobre a Atuação Médica em suas Áreas Durante a Pandemia por COVID-19

**DOI:** 10.36660/abc.20200797

**Published:** 2020-08-19

**Authors:** Gabriel Blacher Grossman, Carlos Alberto Cyrillo Sellera, Carlos Alberto Cordeiro Hossri, Lara Terra F Carreira, Antônio Carlos Avanza, Pedro Ferreira de Albuquerque, Mauricio Milani, Luiz Eduardo Mastrocola, Luiz Eduardo Fonteles Ritt, Odilon Gariglio Alvarenga de Freitas, Tales de Carvalho, William Azem Chalela, Nabil Ghorayeb, Romeu Sergio Meneghelo, Mauricio Batista Nunes, Salvador Manoel Serra

**Affiliations:** 1 Hospital Moinhos de Vento Porto Alegre RS Brasil Hospital Moinhos de Vento, Porto Alegre, RS – Brasil; 2 Clínica Cardionuclear Porto Alegre RS Brasil Clínica Cardionuclear, Porto Alegre, RS – Brasil; 3 Santa Casa de Misericórdia de Santos Santos SP Brasil Santa Casa de Misericórdia de Santos, Santos, SP – Brasil; 4 Faculdade de Medicina Universidade Metropolitana de Santos Santos SP Brasil Faculdade de Medicina da Universidade Metropolitana de Santos, Santos, SP – Brasil; 5 Hospital do Coração São Paulo SP Brasil Hospital do Coração (Hcor), São Paulo, SP – Brasil; 6 Instituto Dante Pazzanese de Cardiologia São Paulo SP Brasil Instituto Dante Pazzanese de Cardiologia,São Paulo, SP – Brasil; 7 Hospital Nossa Senhora do Pilar Curitiba PR Brasil Hospital Nossa Senhora do Pilar, Curitiba, PR – Brasil; 8 Cardiologia Nuclear Curitiba Curitiba PR Brasil Cardiologia Nuclear Curitiba, Curitiba, PR – Brasil; 9 Universidade Vila Velha Vitória ES Brasil Universidade Vila Velha, Vitória, ES – Brasil; 10 Clínica Centrocor Vitória ES Brasil Clínica Centrocor, Vitória, ES – Brasil; 11 Universidade Federal de Alagoas Maceió AL Brasil Universidade Federal de Alagoas, Maceió, AL – Brasil; 12 Hospital Memorial Arthur Ramos Maceió AL Brasil Hospital Memorial Arthur Ramos, Maceió, AL – Brasil; 13 Clínica Fitcordis Brasília DF Brasil Clínica Fitcordis, Brasília, DF – Brasil; 14 Escola Bahiana de Medicina e Saúde Pública Salvador BA Brasil Escola Bahiana de Medicina e Saúde Pública, Salvador, BA – Brasil; 15 Hospital Cardiopulmonar Salvador BA Brasil Hospital Cardiopulmonar, Salvador, BA – Brasil; 16 Minascor Centro Médico Belo Horizonte MG Brasil Minascor Centro Médico, Belo Horizonte, MG – Brasil; 17 Clínica de Prevenção e Reabilitação Cardiosport Florianópolis SC Brasil Clínica de Prevenção e Reabilitação Cardiosport, Florianópolis, SC – Brasil; 18 Universidade do Estado de Santa Catarina Florianópolis SC Brasil Universidade do Estado de Santa Catarina, Florianópolis, SC – Brasil; 19 Hospital das Clínicas Faculdade de Medicina Universidade de São Paulo São Paulo SP Brasil Instituto do Coração (Incor) do Hospital das Clínicas da Faculdade de Medicina da Universidade de São Paulo (HCFM-USP),São Paulo, SP – Brasil; 20 Sociedade Beneficente de Senhoras Hospital Sírio-Libanês São Paulo SP Brasil Sociedade Beneficente de Senhoras Hospital Sírio-Libanês, São Paulo, SP – Brasil; 21 Hospital Israelita Albert Einstein São Paulo SP Brasil Hospital Israelita Albert Einstein, São Paulo, SP – Brasil; 22 Hospital Português Salvador BA Brasil Hospital Português, Salvador, BA – Brasil; 23 Centro de Cardiologia do Exercício Instituto Estadual de Cardiologia Aloysio de Castro Rio de Janeiro RJ Brasil Centro de Cardiologia do Exercício do Instituto Estadual de Cardiologia Aloysio de Castro (CCEx/IECAC), Rio de Janeiro, RJ – Brasil

**Table:** 

Declaração de potencial conflito de interesses dos autores/colaboradores do Posicionamento do Departamento de Ergometria, Exercício, Cardiologia Nuclear e Reabilitação Cardiovascular (DERC/SBC) sobre a Atuação Médica em suas Áreas Durante a Pandemia por COVID-19 Se nos últimos 3 anos o autor/colaborador do Posicionamento:							
Nomes Integrantes do Posicionamento	Participou de estudos clínicos e/ou experimentais subvencionados pela indústria farmacêutica ou de equipamentos relacionados ao posicionamento em questão	Foi palestrante em eventos ou atividades patrocinadas pela indústria relacionados ao posicionamento em questão	Foi (é) membro do conselho consultivo ou diretivo da indústria farmacêutica ou de equipamentos	Participou de comitês normativos de estudos científicos patrocinados pela indústria	Recebeu auxílio pessoal ou institucional da indústria	Elaborou textos científicos em periódicos patrocinados pela indústria	Tem ações da indústria
Antônio Carlos Avanza Jr.	Não	Não	Não	Não	Não	Não	Não
Carlos Alberto Cordeiro Hossri	Não	Não	Não	Não	Não	Não	Não
Carlos Alberto Cyrillo Sellera	Não	Não	Não	Não	Não	Não	Não
Gabriel Blacher Grossman	Não	Não	Não	Não	Não	Não	Não
Lara Terra F. Carreira	Não	Não	Não	Não	Não	Não	Não
Luiz Eduardo Fonteles Ritt	Não	Não	Não	Não	Não	Não	Não
Luiz Eduardo Mastrocola	Não	Não	Não	Não	Não	Não	Não
Mauricio Batista Nunes	Não	Não	Não	Não	Não	Não	Não
Mauricio Milani	Não	Não	Não	Não	Não	Não	Não
Nabil Ghorayeb	Não	Não	Não	Não	Não	Não	Não
Odilon Gariglio Alvarenga de Freitas	Não	Não	Não	Não	Não	Não	Não
Pedro Ferreira de Albuquerque	Não	Não	Não	Não	Não	Não	Não
Romeu Sergio Meneghelo	Não	Não	Não	Não	Não	Não	Não
Salvador Manoel Serra	Não	Não	Não	Não	Não	Não	Não
Tales de Carvalho	Não	Não	Não	Não	Não	Não	Não
William Azem Chalela	Não	Não	Não	Não	Não	Não	Não

## 1. Regras Gerais

- O Departamento de Ergometria, Exercício, Cardiologia Nuclear e Reabilitação Cardiovascular (DERC) acompanha atentamente a pandemia por COVID-19 e suas consequências, encontrando-se alinhado com a Associação Médica Brasileira (AMB), com as posições publicadas pelos departamentos especializados e sociedades filiadas.
- Reconhece que a contenção da pandemia é de estratégia fundamental.
- Este documento reúne de forma atualizada as recomendações para minimizar os riscos dos pacientes e a exposição dos executores durante esse período pandêmico.
- Dada a dinâmica da pandemia, qualquer dessas recomendações poderá ser atualizada caso surjam novos fatos e evidências científicas.
- Todas as medidas preventivas orientadas pelo Ministério da Saúde (MS) e Organização Mundial de Saúde (OMS) deverão ser adotadas de forma sistemática com cuidados de alta qualidade para pacientes com doenças cardiovasculares, por serem considerados de elevados riscos.
- Todo e qualquer procedimento deve respeitar as normas preconizadas de higienização, uso de equipamento de proteção individual (EPI) e restrição de contatos.
- Eventual suspensão, continuação ou interrupção de atividades inerentes às áreas do SBC/DERC devem observar as determinações das autoridades sanitárias locais ou as normas internas das instituições de saúde.
- A remuneração dos exames de teste ergométrico (TE), teste cardiopulmonar de exercício (TCPE), cardiologia nuclear e reabilitação cardiopulmonar, no momento, não poderá sofrer redução em decorrência das medidas tomadas pela pandemia, salvo orientação em contrário da Câmara Técnica Permanente da Classificação Brasileira Hierarquizada de Procedimentos Médicos da AMB.

## 2. Teste Ergométrico e Teste Cardiopulmonar de Exercício

- Avaliação cuidadosa de queixas e sintomas respiratórios e de outros quadros infecciosos agudos desde o contato telefônico para marcação e na confirmação ou não do exame, de modo a evitar a saída desnecessária dos pacientes em locais onde haja distanciamento social. Evitar a vinda de acompanhantes, exceto nos exames de menores de 18 anos e incapazes.^1 , 2^
- O paciente que já tiver apresentado COVID-19 e encontrar-se recuperado, assintomático e estável clinicamente deve postergar a realização do TE e TCPE por, no mínimo, 30 dias após o quadro. Mesmo após a COVID-19, o paciente deverá seguir todas as recomendações e procedimentos descritos neste documento.
- Considerando os riscos potenciais de geração de contaminantes durante TE e TCPE, recomendamos reduzir o número de exames o máximo possível – idealmente, um por hora por ergômetro.
- Após a confirmação do agendamento, orientar os pacientes para virem com as roupas e calçados adequados, pois não haverá possibilidade de utilização de vestiários nas clínicas e hospitais.
- Na chegada do paciente, realizar a reavaliação dos sintomas (preenchimento de questionário epidemiológico específico ou entrevista). Verificar a temperatura corporal além de fornecer máscara cirúrgica logo na entrada do serviço de saúde. As recepcionistas e secretárias deverão usar máscara facial e luvas durante todo o tempo, bem como manter distância segura dos pacientes atendidos.^3 , 4^
- De modo habitual, aplicar termo de consentimento livre e esclarecido, que é obrigatório.

No entanto, sugere-se adição de considerações complementares em função da pandemia vigente: não é possível precisar acuradamente quais os riscos quantitativos de adquirir o coronavírus em um TE e TCPE, mas:

- As medidas preventivas possíveis serão tomadas para minimizar a contaminação.
- Há, provavelmente, maior risco de contrair infecção durante TE e TCPE em relação a um exame fora da pandemia.
- O médico executante deve contextualizar de forma adequada as indicações dos exames e, em casos suspeitos de COVID-19 ou de outra síndrome respiratória aguda (história de febre, tosse, coriza, astenia, taquicardia, cianose, alterações da ausculta pulmonar), comunicar ao médico assistente e suspender a realização do exame.
- A solicitação de TE e TCPE requer, durante a consulta, um exame físico completo do paciente antes da indicação dos mesmos. Devido a essa condição, não é possível a solicitação desses exames através de consultas por telemedicina.^5^
- As salas de realização de exame devem ser amplas e ventiladas. Preferir ambientes com ventilação natural, evitando os sistemas de climatização de ambiente comuns (ventilador e ar-condicionado) devido ao risco potencial destes em dispersar contaminantes no ambiente. ^6^
- Sabe-se que o TE e TCPE são exames de risco teórico de contaminação para o médico executante e equipe executora. Sugere-se que os médicos executores e auxiliares (técnico de enfermagem, paramédicos, enfermeiras) usem, a cada exame, máscara com filtração mínima equivalente à PFF2/N95, óculos de proteção e luvas de procedimentos. Manter o maior tempo possível um distanciamento físico do paciente superior a 2 metros. Sugere-se observar as recomendações institucionais e das secretarias municipais e estaduais da saúde.
- O paciente deve usar máscara com filtração mínima equivalente à PFF1, como as máscaras cirúrgicas, desde sua entrada na área de exames. O paciente deverá higienizar as mãos por meio de lavagem prévia com água e sabão e álcool em gel 70% antes de contato com qualquer superfície e equipamentos existentes na sala de exame.
- Em exames realizados em clínicas e hospitais, antes do início do exame, confirmar realização de higienização e limpeza dos aparelhos e superfícies potencialmente contaminantes. Seguir os protocolos institucionais e que contemplem as recomendações das autoridades sanitárias referentes a esses procedimentos.^6^

No caso de realização de exames em consultórios ou clínicas que não tenham os protocolos instituídos, recomenda-se:

- Fazer limpeza comum do cabo do aparelho de ECG do TE/TCPE com um tecido embebido em álcool a 70%.
- Realizar limpeza e desinfecção para qualquer patógeno transmissível na barra de apoio do ergômetro, tapete da esteira, selim do cicloergômetro, manguito do esfigmomanômetro, estetoscópio e outras superfícies de contato utilizando um ou mais dos produtos recomendados:^1 , 7^
- Com base no hipoclorito de sódio (solução de hipoclorito de sódio ativo a 0,5%).
- Com base em amônia quaternária (QUAT), tomando o cuidado de que a concentração total para o uso deve ser menor que 0,8%.
- Com base no peróxido de hidrogênio acelerado a, no máximo, 0,5%.
- À base de álcool 70% ou álcool e amoníaco quaternário (QUAT).
- Preferencialmente, utilizar materiais descartáveis para realização de TE e TCPE, em especial, quanto aos eletrodos de monitoramento. Descartar todos os materiais de maneira adequada e em local apropriado.
- No caso do TCPE, o médico executante deve confirmar a capacidade efetiva de esterilização de todo o sistema de condução e análise dos gases expirados, além de seguir os protocolos institucionais que contemplem as recomendações das autoridades sanitárias.
- O médico executante deverá se atualizar, verificar e adequar o material de emergência e reanimação cardiorrespiratória, de modo a adequar às novas recomendações para atendimentos de intercorrências e complicações durante a COVID-19.^8 , 9^
- Os serviços de TE e TCPE deverão atualizar seus protocolos de transferência de pacientes em caso de intercorrências e emergências, de acordo com a disponibilidade e orientações dos convênios, cooperativas de saúde e órgãos públicos de socorro.^10^
- Os profissionais (médico executante e auxiliares) com suspeita e diagnóstico de COVID-19 devem ser afastados das atividades, seguirem tratamento e isolamento recomendados.^6^
- Manter os critérios de escolha de ergômetros e protocolos de esforço, os clássicos critérios diagnósticos e prognósticos do TE e do TCPE e as condições pré e pós-analíticas (probabilidades) tradicionalmente utilizadas. Sugerimos descrever no laudo o comportamento do intervalo QT no esforço e no quarto minuto da recuperação. ^11 , 12^
- No momento atual, é razoável considerar o adiamento da realização de TE e TCPE nos casos em que, provavelmente, não impactará diretamente nos cuidados ou nos resultados clínicos nos próximos meses.^3^

## 3. Reabilitação Cardiopulmonar e Metabólica

A COVID-19 tem causado profundo impacto nos serviços de saúde, inclusive nos serviços de reabilitação cardiopulmonar e metabólica (RCPM), fundamentais no manejo clínico dos pacientes com doenças cardiovasculares, pneumopatias e doenças metabólicas, que proporcionam significativas reduções nas taxas de internações hospitalares e mortalidade geral.^13 - 17^

No entanto, no momento, o isolamento social tem sido a pedra angular no controle da COVID-19, especialmente dos pacientes de maior risco para internação hospitalar, complicações respiratórias e mortalidade, que são justamente aqueles com indicação para os programas de RCPM.^18 , 19^ Portanto, em sintonia com as recomendações das autoridades sanitárias mundiais e nacionais, devido ao risco de contágio, os serviços de RCPM com atividades presenciais foram interrompidos.

No contexto da COVID-19, sendo a RCPM imperativa, por exemplo, em processos de recuperação da capacidade funcional de pacientes com insuficiência cardíaca^17 , 20^ ou após eventos e intervenções cardiovasculares, quando o tempo de início dos exercícios após a alta hospitalar pode influenciar na recuperação funcional, controle da doença e redução da mortalidade, consideramos que devem ser priorizados os programas de RCPM a distância, baseados em domicílio, com o apoio do uso de tecnologia digital, que têm sido adotados com bons resultados iniciais por muitos serviços nacionais e internacionais.^17 , 21^

Os exercícios domiciliares devem seguir as recomendações habituais da RCPM convencional, com prescrições individualizadas, sempre que possível baseadas em avaliações prévias.^17 , 21^ Por segurança, orienta-se que, durante os exercícios físicos, seja considerada a escala de percepção de esforço, com recomendação de intensidade leve e/ou moderada. No momento atual, sugerimos que sejam evitados exercícios de alta intensidade, muito desgastantes, com percepção de esforço muito elevado (muito forte).

Ressalta-se que, diante da heterogeneidade nacional da curva epidemiológica dos casos de COVID-19, peculiaridades regionais, incidências de novos casos e necessidades de internações, além de aspectos relacionados com infraestrutura e taxa de ocupação dos serviços de saúde públicos e privados, diferentes recomendações podem ser pertinentes nas localidades, sempre de acordo com as orientações das organizações e autoridades sanitárias.^22^

Assim que sinais de controle da pandemia forem evidentes, havendo maior flexibilização do isolamento social pelas autoridades sanitárias, os serviços convencionais de RCPM com atividades presenciais poderão retomar suas atividades de forma gradual e com a rigorosa observância aos cuidados pertinentes de proteção dos pacientes e profissionais de saúde. Na ocasião do reinício paulatino das atividades, recomendamos:

- Pacientes, profissionais da equipe assistencial e acompanhantes com sintomas gripais ou contato com casos confirmados/suspeitos nos últimos 14 dias devem se manter afastados pelo prazo recomendado pelas organizações e autoridades sanitárias.^23^
- Na triagem dos pacientes que chegam ao serviço, é recomendada a medida de temperatura na região frontal por infravermelho (sem contato cutâneo).
- Uso de máscara facial, álcool em gel e lavagem das mãos com água e sabão são recomendados como obrigatório pelos pacientes e demais frequentadores do ambiente de exercícios, sendo que os profissionais da equipe assistencial devem seguir as determinações dos órgãos de saúde, sindicatos e conselhos profissionais em relação ao uso de EPI.
- Disponibilização de álcool a 70% em *spray* e papéis descartáveis para higienização dos equipamentos de exercícios, antes e após o uso individual, devendo ser evitado o uso compartilhado de equipamentos em circuitos de treinamento (aparelhos de musculação, pesos livres, espaldares e outros).
- Promoção de maior circulação de ar nas salas de exercícios, mantendo, sempre que possível, portas e janelas abertas.
- Redução da quantidade de pacientes atendidos simultaneamente, possibilitando maior distanciamento entre eles.
- Adoção de horários predefinidos de atendimentos, com duração rigorosamente controlada, com intervalos entre as sessões, a fim de evitar a sobreposição de grupos e permitir higienização do ambiente e equipamentos.

Observação: visando à proteção jurídica dos serviços, recomenda-se a solicitação de carta de encaminhamento ao programa de reabilitação do médico assistente, bem como a exigência da assinatura de termo de consentimento após esclarecimento pelos pacientes.

## 4. Cardiologia Nuclear

A orientação para os serviços de cardiologia nuclear durante a pandemia é de que se realizem apenas os estudos urgentes e em pacientes sintomáticos, quando o resultado do exame tiver o potencial de alterar o manejo evolutivo imediato ou que possa impactar o prognóstico do paciente a curto prazo. Também se faz urgente a avaliação de pacientes internados e de pronto atendimento, objetivando direcionar a conduta, reduzir o tempo de internação e expandir a capacidade hospitalar,^3 , 24^

### 4.1. Adaptação da Prática da Cardiologia Nuclear Durante a Pandemia

#### 4.1.1. Considerações Gerais no Agendamento do Exame24,25

- Aumentar o intervalo entre os exames para evitar aglomerações.
- No ato do agendamento, perguntar se o paciente apresenta sinais ou sintomas sugestivos de possível infecção por COVID-19 (febre, tosse, dispneia, fadiga incomum, mialgia, diarreia, anosmia, hiposmia, disgeusia ou ageusia). Em caso afirmativo, de preferência, adiar o agendamento.
- Perguntar se o paciente foi exposto a algum caso confirmado ou suspeito nas 2 semanas anteriores. Em caso afirmativo, de preferência, postergar a marcação do exame.
- Torna-se importante entrar em contato com os pacientes no dia anterior ao exame para garantir que não estejam apresentando sinais ou sintomas suspeitos. Em caso afirmativo, reagendar o exame se possível.
- Os pacientes devem ser instruídos a comparecer ao exame sozinhos. Caso seja necessário o acompanhante, vir com apenas uma pessoa, idealmente sem fatores de risco de relevância como diabetes, cardiopatias não estáveis, arritmias, idosos > 65 anos de idade, entre outros.
- Solicitar para que os pacientes e acompanhantes venham usando EPIs (máscaras faciais como requisito mínimo) ou considerar fornecê-las para serem usadas durante todo o tempo que estiverem no serviço de medicina nuclear.

#### 4.1.2. Considerações no Momento da Chegada do Paciente ao Serviço24-26

- Na chegada ao laboratório nuclear, deve-se questionar novamente o paciente quanto à presença de sintomas e exposição à COVID-19 (por meio do preenchimento de questionário epidemiológico específico e/ou entrevista).
- Dado o risco de transmissão por portadores assintomáticos, a equipe de atendimento ao paciente na sala de espera e demais funcionários não médicos no laboratório devem usar máscara o tempo todo.
- Solicitar para que os pacientes e acompanhantes permaneçam com as máscaras faciais enquanto estiverem no serviço de medicina nuclear.
- As instalações devem garantir que as salas de espera tenham fácil acesso à lavagem das mãos e/ou álcool em gel.
- Manter pelo menos 2 metros de distância entre as pessoas, evitando aglomerações nas salas de espera e instalações do serviço. Orientar que sigam as regras de espaçamento, etiqueta respiratória e lavagem frequente das mãos e/ou álcool gel.
- Evitar a interação entre pacientes internados e ambulatoriais, bem como a de pacientes oncológicos e imunocomprometidos nos casos de serviços que realizem mais de uma modalidade de exame.

#### 4.1.3. Considerações Durante o Exame24-26

***A) Com relação à equipe de trabalho e ambiente***

- Aplicar os princípios gerais de uso de EPIs da saúde durante todo o exame.
- Minimizar o número de funcionários em contato com o paciente.
- Minimizar o tempo de contato paciente/equipe.
- Reforçar a higienização frequente das mãos.
- Se o paciente apresentar sintomas suspeitos, a equipe em contato com ele deverá usar EPI completo (máscara, proteção ocular, avental e luvas) e fornecer uma máscara ao paciente.
- Em pacientes com COVID-19 ativo confirmado, qualquer teste deve ser feito apenas se absolutamente necessário. Consultar as políticas locais de controle de infecção e considerar agendamento como último estudo do dia e em equipamento separado, se possível. Após o exame, uma limpeza terminal completa deve ser realizada na sala e equipamentos.
- *Gantry* , maca, esteira, equipamentos de pressão arterial, estetoscópio e bombas de infusão devem ser limpos após cada exame por pessoal com EPI apropriado.
- É mandatória a realização de limpeza regular das superfícies de contato incluindo maçanetas, superfícies de mesa, computadores, teclados, telefones e equipamentos de ditado por funcionário usando EPI apropriado.

***B) Seleção do protocolo de exame24***

- Selecionar o protocolo de menor duração.
- Considerar protocolos de imagem de um dia.

***C) Seleção do protocolo de estresse*** ^24^

- Como o vírus SARS-CoV-2 é transmitido por gotículas, os procedimentos que envolvem a produção de gotículas ou aerossóis são considerados de maior risco. Sendo assim, o estresse farmacológico é preferido ao TE.
- Se o TE for considerado necessário, a equipe deve usar os EPIs (preferencialmente, máscara N95/PFF2) e manter distância do paciente quando não estiver prestando a assistência direta ou injetando o radiofármaco – seguir as orientações deste documento quanto à realização do TE.
- Manguitos automáticos para medida da pressão arterial devem ser considerados.

***D) Interpretação do exame*** ^24 - 26^

- Evitar vários médicos e/ou estagiários no mesmo local, se possível.
- Nos exames em que se realiza a tomografia computadorizada para a correção de atenuação, as imagens devem ser interpretadas no contexto de possíveis achados pulmonares de COVID-19.

## 5. Cardiologia do Esporte

### 5.1. Atividade Físico-Esportiva na Pandemia por COVID-19

A atividade física regular é essencial para promoção de saúde e correção dos fatores de risco para doenças cardiovasculares, e o sedentarismo piora a evolução e aumenta a mortalidade das doenças cronicodegenerativas. Tanto para o isolamento compulsório como no caso da haver liberação sanitária para as pessoas se deslocarem livremente, listamos orientações para prática de atividade física em domicílio, para academias, em ambientes ao ar livre e esportes em geral.^27^

### 5.2. Atividade Física em Residência

De forma geral, as seguintes orientações devem ser seguidas:^27^

- Praticar exercícios em locais ventilados, mantendo, sempre que possível, janelas e portas abertas.
- Se mais de uma pessoa for se exercitar no mesmo ambiente, manter distância mínima de 2 metros, ou seja, uma pessoa a cada 4 m^2^ .
- De preferência, a prática de atividade física deve ser feita individualmente e, por segurança, executar os exercícios a que esteja habituado.
- Realizar higienização completa com água e sabão ou álcool em gel (70%) das mãos e equipamentos durante a atividade física.
- Usar e trocar toalhas individuais descartáveis ou de tecido.
- Controlar o esforço dispendido nos treinos, com as recomendações previamente estabelecidas pelo seu médico, evitando excessos físicos.
- Suspender os exercícios caso surja algum sintoma: cansaço, dor no peito ou nas costas, tonturas, palpitações, dores musculares, febre, náuseas, vômitos, diarreia ou outros sintomas de estado gripal.Sedentários ou destreinados há muito tempo das atividades físicas só devem realizar atividades físicas leves.

### 5.3. Atividade Física ao Ar Livre

Obrigatório seguir as orientações da autoridade de saúde local quanto às restrições da prática ao ar livre.^27^ Onde as medidas de restrição forem reduzidas, recomenda-se a forma individualizada e isolada, com os devidos cuidados antes referidos. Devemos ter em mente que, no curto prazo, não há tratamento específico para o vírus, e algumas medidas de cuidado devem ser mantidas.

Atualmente, não existem muitos padrões validados de recomendações específicas para a prática de atividade ao ar livre em uma pandemia. Apenas um estudo belgo-holandês sugeriu que a distância de 2 metros é ineficaz para evitar a propagação do vírus, e sugere:

1. Distância de 4 a 5 metros, a ser obedecida entre as pessoas que andam uma atrás da outra.
2. Distância de 10 metros, ao correr ou andar de bicicleta lentamente.
3. Distância de 20 metros, ao andar de bicicleta rapidamente.

Devemos ressaltar que as medidas adotadas, assim como as condutas sugeridas, sofrem constantes mudanças de acordo com o cenário da pandemia.^28 - 30^

### 5.4. Atividades Físicas em Academias

- Disponibilizar álcool em gel a 70% e máscaras faciais para uso dos alunos e colaboradores em todas as áreas da academia (recepção, musculação, peso livre, salas de coletivas, piscina, vestiários, etc.).
- Sugere-se controle ativo de temperatura na entrada da academia.
- Limpeza geral e desinfecção dos ambientes por 30 minutos, uma a duas vezes/dia.
- *Kits* de limpeza em pontos estratégicos das áreas de musculação e peso livre, contendo toalhas de papel para descarte imediato após uso e produto específico de higienização dos equipamentos de treino: colchonetes, halteres e máquinas.
- Limitar a quantidade de alunos na academia e o espaço em que cada aluno deve se exercitar, nas áreas de pesos livres e nas salas de atividades coletivas onde a ocupação simultânea será a cada 4 m^2^ (p. ex., áreas de treino, vestiário).
- Deixar o espaçamento de um equipamento sem uso para o outro.
- Liberar a saída de água no bebedouro somente para uso de garrafas próprias.
- Academias dos condomínios/residenciais: sendo liberadas pelas autoridades sanitárias, recomenda-se reservar horário exclusivo para os moradores da mesma unidade habitacional. Após o uso, é obrigatório realizar medidas de limpeza adequadas.^29^

### 5.5. Fui Acometido por COVID-19 – Quando Posso Voltar à Atividade Física?

Qualquer que seja a atividade física regular escolhida, só deve ser reiniciada após negativação da PCR e liberação clínica. As atividades físico-esportivas de qualquer intensidade necessitam da avaliação médica de pré-participação, objetivando diagnóstico de possíveis sequelas.^30 - 34^

### 5.6. Avaliação de Atletas que Foram Acometidos por Infecção por COVID-19

- Atletas com infecção assintomática e presença de anticorpo confirmada.
- Atletas com histórico de doença leve (sem hospitalização) relacionado com COVID-19, confirmado ou suspeito.
- Atletas com histórico de doença moderada a grave (com hospitalização) relacionado com COVID-19, confirmado ou suspeito.
- Atletas com histórico de infecção por COVID-19 (independentemente da gravidade) com lesão miocárdica confirmada por um ou mais dos seguintes exames: alterações no ECG hospitalar, elevação de troponina ultrassensível ou peptídio natriurético, arritmia ou função cardíaca comprometida.

Obrigatório realizar a avaliação pré-participação (APP) com ECG e demais exames complementares de acordo com a avaliação inicial. Sempre que possível, comparar com exames anteriores, com foco para rastrear achados pós-infecciosos persistentes ou novos.

No retorno ao treinamento, no caso de atletas que tenham apresentado alterações em exames cardiológicos quando acometidos por COVID-19, as imagens cardíacas em série serão necessárias, sendo tal retorno gradual. Além disso, devido ao acometimento cardíaco, deve ser acompanhado por um especialista.
