# Supplementary material for: Posicionamento do Departamento de Ergometria, Exercício, Cardiologia Nuclear e Reabilitação Cardiovascular (DERC/SBC) sobre a Atuação Médica em suas Áreas Durante a Pandemia por COVID-19
Source: Arq Bras Cardiol. 2020 Aug 19;115(2):284–91. [Article in Portuguese] doi: 10.36660/abc.20200797 (PMC8384272; doi:10.36660/abc.20200797)
Supplement: Translation

# Translation: Position Statement of the Brazilian Society of Cardiology Department of Exercise Testing, Sports Exercise, Nuclear Cardiology, and Cardiovascular Rehabilitation (DERC/SBC) on Activities Within its Scope of Practice During the COVID-19 Pandemic

**Authors:** Gabriel Blacher Grossman, Carlos Alberto Cyrillo Sellera, Carlos Alberto Cordeiro Hossri, Lara Terra F Carreira, Antônio Carlos Avanza, Pedro Ferreira de Albuquerque, Mauricio Milani, Luiz Eduardo Mastrocola, Luiz Eduardo Fonteles Ritt, Odilon Gariglio Alvarenga de Freitas, Tales de Carvalho, William Azem Chalela, Nabil Ghorayeb, Romeu Sergio Meneghelo, Mauricio Batista Nunes, Salvador Manoel Serra

**Affiliations:** 1 Brazil Hospital Moinhos de Vento, Porto Alegre, RS – Brazil; 2 Brazil Clínica Cardionuclear, Porto Alegre, RS – Brazil; 3 Brazil Santa Casa de Misericórdia de Santos, Santos, SP – Brazil; 4 Brazil Faculdade de Medicina da Universidade Metropolitana de Santos, Santos, SP – Brazil; 5 Brazil Hospital do Coração (Hcor), São Paulo, SP – Brazil; 6 Brazil Instituto Dante Pazzanese de Cardiologia, São Paulo, SP – Brazil; 7 Brazil Hospital Nossa Senhora do Pilar, Curitiba, PR – Brazil; 8 Brazil Cardiologia Nuclear Curitiba, Curitiba, PR – Brazil; 9 Brazil Universidade Vila Velha, Vitória, ES – Brazil; 10 Brazil Clínica Centrocor, Vitória/ES – Brazil; 11 Brazil Universidade Federal de Alagoas, Maceió, AL – Brazil; 12 Brazil Hospital Memorial Arthur Ramos, Maceió, AL – Brazil; 13 Brazil Clínica Fitcordis, Brasília, DF – Brazil; 14 Brazil Escola Bahiana de Medicina e Saúde Pública, Salvador, BA – Brazil; 15 Brazil Hospital Cardiopulmonar, Salvador, BA – Brazil; 16 Brazil Minascor Centro Médico, Belo Horizonte, MG – Brazil; 17 Brazil Clínica de Prevenção e Reabilitação Cardiosport, Florianópolis, SC – Brazil; 18 Brazil Universidade do Estado de Santa Catarina, Florianópolis, SC – Brazil; 19 Brazil Instituto do Coração (Incor) do Hospital das Clínicas da Faculdade de Medicina da Universidade de São Paulo (HCFM-USP), São Paulo, SP – Brazil; 20 Brazil Sociedade Beneficente de Senhoras Hospital Sírio-Libanês, São Paulo, SP – Brazil; 21 Brazil Hospital Israelita Albert Einstein, São Paulo, SP – Brazil; 22 Brazil Hospital Português, Salvador, BA – Brazil; 23 Brazil Centro de Cardiologia do Exercício do Instituto Estadual de Cardiologia Aloysio de Castro (CCEx/IECAC), Rio de Janeiro, RJ – Brazil

**Table:** 

Declaration of potential conflict of interests of authors/collaborators of the Position Statement of the Brazilian Society of Cardiology Department of Exercise Testing, Sports Exercise, Nuclear Cardiology, and Cardiovascular Rehabilitation (DERC/SBC) on Activities Within its Scope of Practice During the COVID-19 Pandemic If, within the last 3 years, the author/collaborator of the statement:							
Names of statement collaborators	Participated in clinical and/or experimental studies sponsored by pharmaceutical or equipment companies related to this statement	Spoke at events or activities sponsored by industry related to this statement	Was (is) a member of a board of advisors or a board of directors of a pharmaceutical or equipment industry	Participated in normative committees of scientific research sponsored by industry	Received personal or institutional funding from industry	Wrote scientific papers in journals sponsored by industry	Owns stocks in industry
Antônio Carlos Avanza Jr.	No	No	No	No	No	No	No
Carlos Alberto Cordeiro Hossri	No	No	No	No	No	No	No
Carlos Alberto Cyrillo Sellera	No	No	No	No	No	No	No
Gabriel Blacher Grossman	No	No	No	No	No	No	No
Lara Terra F. Carreira	No	No	No	No	No	No	No
Luiz Eduardo Fonteles Ritt	No	No	No	No	No	No	No
Luiz Eduardo Mastrocola	No	No	No	No	No	No	No
Mauricio Batista Nunes	No	No	No	No	No	No	No
Mauricio Milani	No	No	No	No	No	No	No
Nabil Ghorayeb	No	No	No	No	No	No	No
Odilon Gariglio Alvarenga de Freitas	No	No	No	No	No	No	No
Pedro Ferreira de Albuquerque	No	No	No	No	No	No	No
Romeu Sergio Meneghelo	No	No	No	No	No	No	No
Salvador Manoel Serra	No	No	No	No	No	No	No
Tales de Carvalho	No	No	No	No	No	No	No
William Azem Chalela	No	No	No	No	No	No	No

## 1. General Rules

- The Brazilian Society of Cardiology Department of Exercise Testing, Sports Exercise, Nuclear Cardiology, and Cardiovascular Rehabilitation (DERC) is closely monitoring the COVID-19 pandemic and its consequences, and is aligned with the Brazilian Medical Association (AMB) regarding the position statements issued by its specialized departments and affiliate societies.
- It recognizes that containment of the pandemic is a core strategy.
- This document provides an up-to-date overview of recommendations to minimize risks to both patients and clinicians during this pandemic period.
- Given the dynamics of the pandemic, any of these recommendations may be updated if and as new facts and scientific evidence arise.
- All preventive measures advised by the Ministry of Health and the World Health Organization (WHO) should be systematically incorporated into high-quality care for patients with cardiovascular diseases, as they are considered to be at high risk.
- Any and all procedures must follow recommended standards for disinfection, use of personal protective equipment (PPE), and contact precautions.
- Any discontinuation, continuation, or interruption of activities inherent to the SBC/DERC scope of practice must comply with the provisions of local health authorities or the bylaws of the health facilities where they are conducted.
- At this time, compensation for treadmill exercise tests (TMETs), cardiopulmonary exercise tests (CPETs), nuclear cardiology procedures, and cardiopulmonary rehabilitation cannot be reduced as a result of any measures taken in the fight against the pandemic, unless otherwise instructed by the AMB Permanent Technical Committee on the Hierarchical Brazilian Classification of Medical Procedures.

## 2. Treadmill Exercise Test and Cardiopulmonary Exercise Test

- Careful assessment of respiratory symptoms and complaints and other acute infectious conditions should begin when the patient calls to schedule the test. The appointment may then be confirmed or discouraged, so as to avoid unnecessary movement of patients where social distancing measures are in place. Patients should come alone, except for children (under 18) and those otherwise incapable.^1 , 2^
- Patient who have already contracted COVID-19 and have recovered, if asymptomatic and clinically stable, should postpone TMET and CPET for at least 30 days after recovery. Even patients recovered from COVID-19 must follow the recommendations and procedures described herein.
- Considering the potential risk for generation of contaminants during TMET and CPET, we recommend that the number of tests be reduced as much as possible—optimally, one test per hour per ergometer.
- Once the appointment has been confirmed, instruct patients to come already wearing appropriate clothing and footwear, as they will not be allowed to use changing rooms at the clinic or hospital.
- Upon arrival, reassess the patient for signs and symptoms of COVID-19 by interview or completion of a specific epidemiological questionnaire. Measure body temperature and provide a surgical mask at the entrance to the facility. Receptionists and other secretarial staff must wear a face mask and gloves at all times, as well as maintain a safe distance from patients.^3 , 4^
- As usual, all patients must sign an informed consent form. This is mandatory.

However, it is suggested that additional considerations be included in the form due to the ongoing pandemic, namely: it is impossible to accurately specify the quantitative risk of contracting the coronavirus during a TMET or CPET, but:

- All possible preventive measures will be undertaken to minimize contamination.
- The risk of contracting an infection during a TMET or CPET is probably higher compared to that of the same test performed once the pandemic is over.
- The physician in charge of the test must adequately contextualize the indications for the test and, in case COVID-19 or any other acute respiratory syndrome is suspected (history of fever, cough, nasal discharge, weakness, tachycardia, cyanosis, abnormal pulmonary auscultation), inform the attending physician and discontinue the test.
- An order for a TMET or CPET must be preceded by a thorough physical examination of the patient to determine whether the test is truly indicated. Thus, such tests cannot be ordered or requested via telemedicine.^5^
- Examination rooms should be large and well-ventilated. Natural ventilation is preferred; common climate-control systems (fans and HVAC) should be avoided due to the potential for environmental dispersal of contaminants.^6^
- It is well established that TMET and CPET pose a theoretical risk of contamination for the performing clinician and team. The physician and auxiliary staff (technicians, paramedics, nurses) are advised to wear a respirator (FFP2/N95-equivalent filtration efficiency or higher), goggles, and procedure gloves throughout the test. Staff are to remain at least 2 meters away from the patient for as long as possible. Institutional recommendations and the advice of municipal and state health departments should be followed.
- Patients must wear an FFP1-equivalent medical mask (such as a surgical mask) upon entering the testing area of the facility. Patients must wash their hands with soap and water and apply hand sanitizer (containing at least 70% alcohol) before contact with any equipment or any other surface in the examination room.
- In the clinic and hospital setting, before the test begins, staff are to confirm that potentially contaminating devices and surfaces have been properly cleaned and sanitized. Institutional protocols should incorporate the recommendations of health authorities regarding these procedures and must be followed.^6^

If a test is to be performed in an office setting or at a clinic that has no environmental services protocols in place, the following actions are recommended:

- Clean the ECG cables with a 70% alcohol wipe.
- Clean and perform high-level disinfection for transmissible pathogens of the ergometer support bar, treadmill mat, cycle ergometer saddle, sphygmomanometer cuff, stethoscope, and other high-touch surfaces using one or more of the following recommended products:^1 , 7^
- Active chlorine-based (0.5% sodium hypochlorite solution).
- Quaternary ammonium (“quat”)-based (final concentration no higher than 0.8%).
- Accelerated hydrogen peroxide-based (concentration no higher than 0.5%).
- Alcohol-based (concentration no lower than 70%) or alcohol plus a quaternary ammonium (“quat”) compound.
- Disposable materials—especially monitoring electrodes—are preferred when performing TMET and CPET. Dispose of all materials properly and in an appropriate container.
- In case of CPET, the physician in charge of the test must confirm there is capacity to sterilize the entire system effectively, including the expired gases circuit and analyzer, and that institutional protocols which incorporate the recommendations of health authorities are followed.
- The physician in charge of the test is to assess and update the crash cart/trolley and all other emergency equipment so as to ensure it is adapted to the latest recommendations for resuscitation and treatment of complications during the COVID-19 pandemic.^8 , 9^
- Facilities which offer TMET and CPET are to update their protocols for patient transfer in the event of complications and emergencies in concert with the availability and guidelines of insurers, health management organizations, and local emergency medical services.^10^
- Professionals (including the physician and auxiliary staff) with a suspected or confirmed diagnosis of COVID-19 are to be relieved of their duties and follow current recommendations for treatment and self-isolation.^6^
- The usual criteria for selection of ergometers and exercise protocols, the classic diagnostic and prognostic criteria for TMET and CPET, and the conventional pre- and post-test probabilities still apply. We suggest that the test report describe the behavior of the QT interval during exertion and at the fourth minute of recovery.^11 , 12^
- At the present time, it is reasonable to consider postponing TMET and CPET whenever the test is unlikely to have a direct impact on care or clinical outcome in the following months.^3^

## 3. Cardiopulmonary and Metabolic Rehabilitation

The COVID-19 pandemic has had a profound impact on health services, including cardiopulmonary and metabolic rehabilitation (CPMR) services, which play a fundamental role in the clinical management of patients with cardiovascular, pulmonary, and metabolic diseases, providing significant reductions in hospitalization and overall mortality rates.^13 - 17^

However, to date, isolation and social distancing have been the cornerstone of COVID-19 control, especially for patients at higher risk of hospitalization, respiratory complications, and mortality—precisely those with indications for CPMR programs.^18 , 19^ Therefore, in line with the recommendations of global and national health authorities, CMR services involving face-to-face activities have been suspended due to the risk of contagion.

Within the context of COVID-19, considering that CPMR is essential for the process of recovery of functional capacity of patients with heart failure^17 , 20^ or after cardiovascular events and interventions, and that the time to initiation of an exercise program after hospital discharge can influence functional recovery, disease management, and mortality rates, we believe that home-based CPMR programs—delivered at a distance with the support of digital technologies—should be prioritized. Such programs have been adopted to good effect by many services in Brazil and elsewhere.^17 , 21^

Home-based exercises should follow the usual recommendations for conventional CPMR. The exercise prescription should be individualized and based on prior evaluation whenever possible.^17 , 21^ For safety purposes, it is recommended that the scale of perceived exertion be used during physical exercise, which should be of light and/or moderate intensity at most. At the present time, we suggest that high-intensity, exhausting exercises with a very high rating of perceived exertion be avoided.

It bears stressing that, given the nationwide heterogeneity of the epidemiological curve of COVID-19 and regional differences in the incidence of new cases, hospitalization rates, and infrastructure (such as the occupancy rate of public and private hospitals), different recommendations may be relevant to different locations. Therefore, these should always follow the guidance of health organizations and authorities.^22^

Once there is clear evidence that the pandemic is being brought under control and social isolation measures are lifted by health authorities, conventional CPMR services (i.e., including face-to-face activities) will be able to resume their activities, gradually and with strict observance of the relevant precautions for the protection of patients and providers alike. As activities are gradually resumed, the following recommendations will apply:

- Patients, their chaperones, and staff members with flu-like symptoms or a history of contact with confirmed or suspected cases in the preceding 14 days are to self-isolate for however long is recommended by health organizations and local health authorities.^23^
- Noncontact (infrared) temperature screening of patients upon arrival is advised.
- Face coverings, hand sanitizer, and frequent handwashing with soap and water are mandatory for patients and all others who attend exercise facilities. Staff members are to follow the recommendations of health regulatory agencies, trade unions, and relevant professional boards/trade associations regarding the use of PPE.
- Spray bottles containing 70% alcohol and disposable paper towels are to be made available for disinfection of exercise equipment before and after individual use. Shared use of equipment (weight machines, weight benches, free weights, etc.) should be avoided.
- To promote increased air circulation, keep doors and windows open whenever possible.
- Reduce the number of patients allowed in the facility simultaneously, so as to allow greater distancing between them.
- Implement predefined working hours, with a strictly controlled duration of and intervals between sessions, to overlap between groups and allow disinfection of the environment and equipment.

Note: to provide a measure of legal protection to facilities that offer CPMR, it is recommended that a letter of referral for rehabilitation be obtained from the patient’s attending physician, as well as written informed consent from patients themselves.

## 4. Nuclear Cardiology

During the pandemic, nuclear cardiology services are advised to limit their activities to urgent studies in symptomatic patients, in which the test result has the potential to change immediate management or affect the patient’s short-term prognosis. It is also essential that scans be performed on inpatients and emergency department patients requiring urgent assessment, as this can guide management, shorten hospital stay, and thus free up hospital capacity.^3 , 24^

### 4.1. Adaptation of Nuclear Cardiology Practices During the COVID-19 Pandemic

#### 4.1.1. General Considerations When Scheduling a Nuclear Cardiology Scan24,25

- Increase the interval between scans to avoid crowding.
- When scheduling, ask if the patient has any signs or symptoms suggestive of possible COVID-19 infection (fever, cough, dyspnea, unusual fatigue, myalgia, diarrhea, anosmia, hyposmia, dysgeusia, or ageusia). If so, the appointment should preferably be postponed.
- Ask if the patient has been exposed to a confirmed or suspected case in the preceding 2 weeks. If so, the scan should preferably be postponed.
- Patients should be contacted the day before the scan to ensure they are not experiencing any suspicious signs or symptoms. If so, the scan should be rescheduled if possible.
- Patients should be instructed to come in for the scan alone. Patients who absolutely require a companion or chaperone should come with only one person, ideally someone with no relevant risk factors (such as diabetes, unstable heart disease, arrhythmias, age >65 years, etc.).
- Ask that patients and their chaperones come in already wearing PPE (face coverings at the very least). Alternatively, the facility should consider providing PPE to be worn for the entire time patients are at the nuclear medicine department.

#### 4.1.2. Considerations Upon Patient Arrival at the Facility24-26

- Upon arrival at the nuclear cardiology lab, reassess the patient for the presence of signs, symptoms, or potential exposure to COVID-19, by interview or completion of a specific epidemiological questionnaire.
- Given the risk of transmission by asymptomatic carriers, patient care staff in the waiting room and all other non-medical staff in the laboratory are to wear a mask at all times.
- Instruct patients and their chaperones to wear face coverings while in the nuclear medicine department.
- Facilities must ensure that waiting rooms have easy access to hand washing stations and/or hand sanitizer.
- Enforce a distance of at least 2 meters between individuals, so as to avoid crowding in waiting rooms and other facilities. Instruct all those who attend to follow distancing rules, respiratory etiquette, and frequent hand washing and/or application of hand sanitizer.
- In facilities offering modalities other than just nuclear cardiology, interactions between inpatients and outpatients should be avoided, as should any contact between outpatients and patients with cancer or other immunocompromised patients.

#### 4.1.3. Considerations During the Scan24-26

***A) Regarding staff and the environment***

- General principles of PPE use apply throughout the scan.
- Minimize the number of staff members in contact with the patient.
- Minimize patient–staff contact time.
- Highlight importance of frequent hand hygiene.
- If the patient has suspicious symptoms, all staff members in contact with the patient must wear full PPE (respirator, eye protection, apron, and gloves) and provide a mask to the patient.
- In patients with confirmed active COVID-19, scans should be performed only if absolutely necessary. Check local infection control policies and consider scheduling these patients as the last scan of the day. Use a separate scanner if possible. After the scan, complete terminal cleaning of the room and all equipment is to be performed.
- The scanner gantry, bed, gurney, treadmill, sphygmomanometer, stethoscope, and infusion pumps are to be cleaned after each scan by personnel wearing appropriate PPE.
- Regular cleaning of high-touch surfaces (including door handles, tables and desks, computers, keyboards, telephone receivers, and dictation equipment) by personnel wearing appropriate PPE is mandatory.

***B) Selection of scan protocol*** ^24^

- Choose the shortest protocol.
- Consider one-day imaging protocols.

***C) Selection of stress protocol*** ^24^

- As the SARS-CoV-2 virus is spread by droplets, procedures which generate droplets or aerosols are considered to pose the greatest risk. Therefore, pharmacological stress tests are preferred to exercise tests.
- If an exercise test is considered absolutely necessary, the staff must wear appropriate PPE (preferably, an N95/FFP2 respirator) and keep their distance from the patient except if providing direct care or while injecting the tracer. Follow the guidelines of this position statement regarding TMET.
- Use of automated blood pressure cuffs in lieu of sphygmomanometers requiring operator intervention should be considered.

***D) Interpretation of test results*** ^24 - 26^

- Avoid the presence of several physicians and/or interns in the same location if possible.
- In scans requiring computed tomography (CT)-based attenuation correction, CT images must be interpreted in the context of pulmonary findings possibly indicative of COVID-19.

## 5. Sports Cardiology

### 5.1. Physical Activity and Sports During the COVID-19 Pandemic

Regular physical activity is essential for the promotion of health and correction of risk factors for cardiovascular diseases. A sedentary lifestyle worsens the natural history of chronic degenerative diseases and increases mortality. Both while lockdown measures are in place and once restrictions on mobility have been lifted, the following guidance applies for physical activity at home, in gyms and health clubs, and outdoors, as well as for participation in sports in general.^27^

### 5.2. Physical Activity at Home

Broadly, the following guidelines are to be followed:^27^

- Exercise in a well-ventilated place; keep doors and windows open whenever possible.
- If more than one person will exercise in the same room, keep a minimum distance of 2 meters between them (i.e., one person per 4 m^2^ ).
- Preferably, physical activities should be practiced individually. To increase safety, stick to those exercises which you already used to performing.
- Wash hands and exercise equipment very well with soap and water or hand sanitizer (70% alcohol-based) during physical activities.
- Use disposable towels or individual fabric towels, laundering them after every use.
- Do not overexert yourself while training; follow your physician’s advice.
- Stop exercising at once if any of the following symptoms appear: fatigue, chest or back pain, dizziness, palpitations, muscle pain, fever, nausea, vomiting, diarrhea, or flu-like symptoms.
- Sedentary individuals and those who have not trained for a long time should limit themselves to light physical activity only.

### 5.3. Outdoor Physical Activity

Follow the guidelines of local health authorities regarding restrictions on outdoor physical activity.^27^ Even where restrictions have already been lifted, individual, isolated exercise is recommended, as described above. Always bear in mind that, as of yet, there is still no specific treatment for the virus, and some precautionary measures must continue to be followed.

There are not many validated standards for specific recommendations regarding the practice of outdoor activities during a pandemic. Only one Belgian–Dutch study suggested that a distance of 2 meters is ineffective in preventing the spread of the virus during such activities. Instead, the authors suggest:

1. A distance of 4 to 5 meters between people walking behind one another.
2. A distance of 10 meters when jogging or cycling slowly.
3. A distance of 20 meters when cycling quickly.

It bears stressing that the aforementioned measures and suggested behaviors are subject to constant change as the pandemic evolves.^28 - 30^

### 5.4. Physical Activity in Gyms and Fitness Clubs

- Hand sanitizer (70% alcohol-based) and face masks should be provided for use by members and staff in all areas (front desk, weight rooms, free weights, classrooms, swimming pool, changing rooms, etc.).
- Active temperature screening at the front door is recommended.
- All rooms should undergo 30 minutes of general cleaning and disinfection once or twice a day.
- Cleaning kits, containing single-use paper towels for immediate disposal and a specific product for disinfection of equipment (mats, dumbbells, weight machines, etc.), should be placed at strategic points in the weight training and free weight areas.
- Limit the number of members in the gym at any one time and the space allocated to each member. In free-weight areas, classrooms, and other shared spaces (e.g., training areas, locker rooms), occupancy should be limited to one person per 4 m^2^ .
- Use of contiguous machines should not be allowed (i.e., if one machine is in use, the next one over should be out of service).
- Water cooler privileges should be limited to refilling of individual bottles.
- Home and building gyms, once cleared to reopen by the health authorities, should set aside exclusive hours for residents of the same apartment or unit. Proper cleaning after use is mandatory.^29^

### 5.5. I’ve Been Diagnosed with COVID-19 – When Can I Resume Physical Activities?

Whichever regular physical activity is desired, clearance for practice is contingent upon negative PCR and clinical reassessment. Before resuming any physical activity, regardless of intensity, a pre-exercise assessment is mandatory to diagnose potential sequelae of COVID-19.^30 - 34^

### 5.6. Assessment of Athletes Who Have Contracted COVID-19

- Athletes with asymptomatic infection and confirmed presence of antibodies (positive serology).
- Athletes with a history of mild COVID-19-related illness (not requiring hospitalization), confirmed or suspected.
- Athletes with a history of moderate-to-severe COVID-19-related illness (requiring hospitalization), confirmed or suspected.
- Athletes with a history of COVID-19 infection (regardless of severity) with evidence of myocardial injury, confirmed by one or more of the following: in-hospital ECG changes, elevation of troponin or natriuretic peptide levels, arrhythmia, or impairment of heart function.

Pre-exercise assessment with ECG and additional tests (as guided by the initial assessment) is mandatory. Whenever possible, confront with findings of previous tests, with a view to screening for persistent or de novo post-infectious symptoms.

Athletes who exhibited cardiac abnormalities during COVID-19 infection will require serial cardiac imaging before resuming their regular activities, and should resume these activities only gradually. In addition, all patients with cardiac involvement must be followed by a specialist.
